# Efficacy and safety of endovascular coiling vs surgical clipping for patients with ruptured carotid-ophthalmic aneurysm: A protocol for systematic review and meta-analysis

**DOI:** 10.1097/MD.0000000000023235

**Published:** 2020-11-20

**Authors:** Guan-Jun Feng, Feng Gao, Xiao-Yuan Huang, Paer Hati, Xiao-Peng Yang, Hong-Xing Wu

**Affiliations:** Department of Neurosurgery, People's Hospital of Xinjiang Uygur Autonomous Region, Urumqi, China.

**Keywords:** aneurysm, endovascular, meta-analysis, surgical clipping

## Abstract

**Background:**

Carotid-ophthalmic aneurysms are relatively rare, and represent 1% of all intracranial aneurysms. Generally, endovascular coiling and surgical clipping are the 2 most commonly used methods to treat ruptured carotid-ophthalmic aneurysms, it provides the most favorable outcome for a patient. This study aims to assess the efficiency and safety of endovascular coiling vs surgical clipping for patients with a ruptured carotid-ophthalmic aneurysm.

**Methods:**

A comprehensive systematic literature review was done in PubMed, EMBASE, Cochrane Library, Web of Science, Scopus, China National Knowledge Infrastructure (CNKI), and WanFang databases. Only randomized trials that compared endovascular coiling with surgical clipping in patients with ruptured carotid-ophthalmic aneurysm was included. Data was extracted independently by 2 review authors. Moreover, the quality of study and bias risk was evaluated by utilizing an appropriate method. Triallists will be contacted to acquire missing information. The data is presented as risk ratio and mean difference, or standardized mean difference with 95% confidence intervals.

**Results:**

The results from the present research shall be published in a peer-reviewed journal.

**Conclusion:**

The present study summarizes the direct and in-direct evidence to judge the efficiency and safety of these 2 methodologies to treat ruptured carotid-ophthalmic aneurysms and attempt to find the most efficiency and safety therapeutical method.

**Ethics and Dissemination:**

The present study is a meta-analysis based on published evidence. As a result, ethics approval and patient consent are not needed.

## 1 Introduction

Carotid-ophthalmic aneurysms are known to be positioned in the medial or anteromedial wall of the internal carotid artery, between the ophthalmic artery and posterior communicating artery.^[1,2]^ Carotid-ophthalmic aneurysms are comparatively rare, and represent 0.3% to 1% of all intracranial aneurysms, and 0.9% to 6.5% of all internal carotid artery aneurysms.^[3]^ Carotid-ophthalmic aneurysms can result in sight-threatening symptoms. In initial clinical evaluations, it can be misdiagnosed as a disorder in the eye. The carotid-ophthalmic aneurysms enlarges, it advances, and, ultimately, it will lead to fatal subarachnoid hemorrhage. In addition to being devastative for patients, the rupture of intracranial aneurysms is also a disastrous situation for clinicians. However, due to the rarity of the disease, the best therapeutic strategy and technique for ruptured carotid-ophthalmic aneurysm is yet to be established.

Endovascular coiling and surgical clipping are two common forms of treatment for ruptured intracranial aneurysms.^[4–7]^ Reportedly, several studies over the last decade have utilized endovascular coiling and surgical clipping techniques, and presented complications in treating ruptured intracranial aneurysms.^[2,8,9]^ However, a majority of reported studies are small, which makes it challenging to comprehend the complications and clinical outcomes in treating ruptured intracranial aneurysms.^[2,9]^

In order to ascertain the efficacy and safety of endovascular coiling vs surgical clipping for patients with ruptured carotid-ophthalmic aneurysm, a systematic review and meta-analysis is performed on the existing literature to analyze outcomes by different treatment types based on data from published studies.

## 2 Methods

### 2.1 Study registration

This protocol has been registered in the Open Science Framework (OSF, http://osf.io/). This protocol of systematic review and meta-analysis registration DOI number is 10.17605/OSF.IO/E82SP. In addition, we will complete this protocol based on the Preferred Reporting Items for Systematic Review and Meta-Analyses Protocols statement guidelines.^[10]^

## 3 Criteria for considering studies for this review

### 3.1 Types of studies

Randomized controlled trials (RCTs) comparing endovascular coiling with surgical clipping.

### 3.2 Types of participants

The participants of this study included those who were diagnosed with ruptured intracranial arterial aneurysm, and undergoing endovascular coiling or surgical clipping.

### 3.3 Types of interventions

Any RCT study involving endovascular intervention with coils, compared with surgical clipping.

### 3.4 Types of outcome measures

#### 3.4.1 Primary outcomes

Death or dependency in activities of daily living was the primary outcome. A poor clinical outcome was defined as Glasgow Outcome Scale (GOS) one-three or modified Rankin Scale (mRS) three-six.^[11,12]^

#### 3.4.2 Secondary outcomes

(1) Death from any cause;
(2) Novel postsurgical vasospasm or infarction during hospitalization;
(3) Rebleeding;
(4) Intervention-associated complications, defined as a clinical deterioration observed within 24 hours after the intervention;
(5) Postoperative-associated infection within 1 week after surgery, including the lung, the skin, the urinary tract, or the wound.

### 3.5 Search methods

#### 3.5.1 Search resources

Relevant studies were identified in the following electronic databases: PubMed (1966 to 23 September 2020), EMBASE (1980 to 23 September 2020), Cochrane Library (CENTRAL; 2020, Issue 10), Web of Science (1965 to 23 September 2020), Scopus (1823 to 23 September 2020), China National Knowledge Infrastructure (CNKI; last searched 23 September 2020), and WanFang databases (last searched 23 September 2020).

#### 3.5.2 Searching other resources

In attempting to identify additional relevant unpublished, ongoing studies, and articles published elsewhere, the trialists were contacted, the reference lists in all relevant publications were checked, and searched ClinicalTrials.gov (http://clinicaltrials.gov/).

#### 3.5.3 Search strategies

These key terms were utilized in combination to search for articles: “coiling∗ OR coils∗ OR clipping∗ OR endovascular∗ OR surgical∗” AND “aneurysm∗ OR carotid-ophthalmic∗ OR Ophthalmic Artery OR ruptured carotid-ophthalmic aneurysm” AND “randomized∗ OR randomized∗ OR RCT∗ OR RCTs∗.” Language restrictions include English or Chinese.

### 3.6 Data collection and analysis

#### 3.6.1 Selection of studies

**Figure 1:**
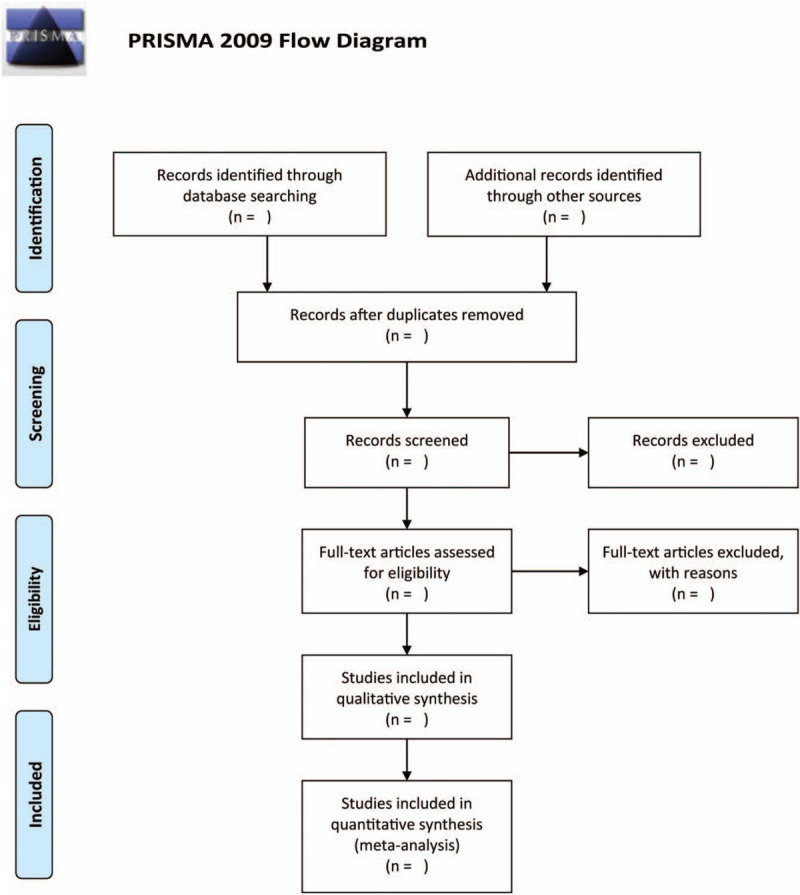
Flow diagram of the literature search.

The studies identified by the search were reviewed independently by 2 review authors to evaluate their relevance by adopting the selection criteria. Any differences in opinion were resolved through discussions via a third review. Figure 1 shows the flowchart of the research.

#### 3.6.2 Data extraction and management

The data will be extracted independently by 2 review authors. We plan on recording the following information into Excel table:

(1) Basic information: author, publication year, age, gender, sample size, time period; and
(2) Characteristic of participants: aneurysm classification, aneurysm size, Hunt and Hess grade, follow-up, intervention method, and clinical outcomes.

#### 3.6.3 Assessment of risk of bias in included studies

The risk of bias in the included studies will be evaluated independently by utilizing Cochrane's tool.^[13]^ The following 7 domains will be evaluated, namely, random sequence generation, allocation concealment, blinding of participants and personnel, blinding of outcome assessment, incomplete outcome data, selective outcome reporting, and other sources bias. Each domain for included trials will be judged as “high risk,” “low risk” or “unclear risk.” It is planned to resolve any disagreements through discussions.

#### 3.6.4 Measures of treatment effect

An estimate of the treatment effect across trials will be calculated (the dichotomous outcomes will be expressed by the risk ratio with 95% confidence intervals (CI), while the continuous outcomes will be expressed by the mean difference or standardized mean difference with 95% CI).

#### 3.6.5 Dealing with missing data

The corresponding authors will be contacted to acquire any missing data. In the case where a response is not received, the trials with incomplete data will be removed, and the reason and impact of missing data will be explained.

#### 3.6.6 Assessment of heterogeneity

The statistical heterogeneity will be assessed using the *Chi-squared* test statistics and *I*^*2*^ statistic.^[14]^ Where *P* < .1 or *I*^*2*^ > 50%, there is substantial heterogeneity. Furthermore, the random-effects model will be used to calculate a weighted estimate of the treatment effects.^[15]^ On the contrary, where *P* > .1 or *I*^*2*^ < 50% indicates that there is no evidence of obvious statistical heterogeneity, and then the fixed-effects model will be used to calculate a weighted estimate of the treatment effects.^[16]^

#### 3.6.7 Assessment of reporting biases

In the case where the number of studies included in the review exceeds 10, the reporting bias shall be investigated by utilizing funnel plots and Egger test.^[17,18]^

#### 3.6.8 Data synthesis

A summarized risk ratio, mean difference, and standardized mean difference will be generated with 95% CI using the fixed-effects or random-effects model meta-analysis provided by the Review Manager software.

#### 3.6.9 Subgroup analysis

Subgroup analysis was not performed in the current study.

#### 3.6.10 Sensitivity analysis

In order to determine the stability and reliability of the findings by excluding studies with low-quality or unclear methodological data.

## 4 Discussion

Aneurysms of the carotid-ophthalmic segment are quite rare, and only a minority of them will rupture. In other words, only very few ruptured carotid-ophthalmic aneurysms were included in the International Subarachnoid Aneurysm Trial study, as a result, there is still no consensus regarding the best form of treatment. To the best knowledge of the author, the present study will be the first systematic review and meta-analysis that compares 2 most commonly used methods to treat ruptured carotid-ophthalmic aneurysms with RCTs. The data from the most recent trials will be synthesized and summarized. The work done in the current study will provide evidence for clinicians to establish optimal treatment strategies for patients with ruptured carotid-ophthalmic aneurysm.

## Author contributions

**Conceptualization:** Hongxing Wu.

**Data curation:** Hongxing Wu.

**Formal analysis:** Feng Gao, Hongxing Wu.

**Funding acquisition:** Guan-Jun Feng, Feng Gao, Hongxing Wu.

**Investigation:** Guan-Jun Feng, Feng Gao, Xiao-Yuan Huang, Paer Hati.

**Methodology:** Guan-Jun Feng, Feng Gao, Xiao-Yuan Huang, Paer Hati, Xiao-Peng Yang.

**Project administration:** Guan-Jun Feng, Feng Gao, Xiao-Yuan Huang, Xiao-Peng Yang.

**Resources:** Guan-Jun Feng, Xiao-Yuan Huang, Paer Hati, Xiao-Peng Yang.

**Supervision:** Xiao-Yuan Huang.

**Validation:** Xiao-Yuan Huang.

**Visualization:** Xiao-Peng Yang.
